# Hygiene practices among food vendors in urban, semi-urban, and rural Ghanaian settings: an ethnographic qualitative study

**DOI:** 10.3389/fpubh.2026.1907787

**Published:** 2026-08-14

**Authors:** Forgive Awo Norvivor, Elijah Kwasi Peprah, Maxwell Boadi Bosompem, Solomon Totime, Beatrice Attah Semanu

**Affiliations:** 1 Fred N. Binka School of Public Health, University of Health and Allied Sciences, Ho, Ghana; 2 Department of Environmental Health and Sanitation, School of Hygiene, Accra, Ghana; 3 Environmental Health and Sanitation Unit, Municipal Assembly, West Akim, Ghana

**Keywords:** food hygiene, food safety, Ghana, qualitative research, street food vendors

## Abstract

**Background:**

Street food vending is an important source of affordable meals and livelihood in Ghana; however, poor hygiene practices among vendors remain a major contributor to foodborne diseases. Despite this public health concern, qualitative evidence from smaller towns and peri-urban communities in Ghana remains limited. This study explored the hygiene practices, knowledge, attitudes, and regulatory experiences of food vendors in Asamankese, Adabraka, and Kpando Gabi.

**Methods:**

A descriptive qualitative research design was employed using structured in-depth interviews and field observations. Twenty food vendors were selected through convenience sampling across the three study sites. Data were analyzed using reflexive thematic analysis.

**Results:**

Four major themes emerged from the analysis: personal hygiene practices, environmental and food handling hygiene, knowledge and perceptions of food hygiene, and enforcement of food safety regulations. Although many vendors reported engaging in routine hygiene practices such as bathing, cleaning vending areas, and washing utensils before work, field observations revealed inconsistencies between reported and actual practices, particularly regarding hand hygiene and the handling of food and money simultaneously. The use of Personal Protective Equipment was inconsistent across study sites. Vendors generally associate food hygiene with visible cleanliness and customer attraction rather than microbial contamination risks. Regulatory inspections were irregular and focused mainly on blood testing and health card renewal, with limited assessment of actual vending conditions.

**Conclusion:**

Improving food hygiene among street food vendors requires more than awareness creation alone. Consistent regulatory enforcement, improved sanitation infrastructure, and practical, context-specific hygiene training are necessary to strengthen food safety practices in informal food vending settings in Ghana.

## Introduction

Street food vending plays a vital role in the daily lives of urban and non-metropolitan settlements in sub-Saharan Africa. In Ghana, as in many low and middle-income countries, street-vended foods offer affordable, ready-to-eat meals to large segments of the population, including workers, students, and low-income households who are unable to prepare meals at home (1, 2). The sector also serves as a major source of income and livelihood for millions of vendors, most of whom are women (2). This widespread reliance on street food on both the demand and supply sides makes the hygiene conditions under which food is prepared and sold a direct and consequential public health issue.

The global burden of foodborne disease provides the epidemiological basis for that concern. The World Health Organization estimates 600 million people get sick from contaminated food each year, including 420,000 deaths and 33 million disability-adjusted life years (DALYs) lost (3, 4). Sub-Saharan Africa has the highest burden per capita in the world, with foodborne diseases causing an estimated 1,200 to 1,300 DALYs/100,000 population in parts of the region, compared with 35 per 100,000 in North America (4, 5). Children aged under five years carry 40 percent of the overall global burden despite representing a much smaller proportion of the global population (3). In Ghana, about 420,000 people are affected by foodborne illness each year, out of which an estimated 65,000 people die from foodborne disease (6). The World Bank estimates that foodborne diseases result in annual productivity losses of approximately US$95.2 billion in low- and middle-income countries, highlighting the substantial economic burden associated with foodborne illnesses in addition to their impact on morbidity and mortality (7).

Street food vendors have been a well-documented source of food-borne illness in low and middle-income countries. Research from Ghana and various West African countries has consistently recognized poor personal hygiene, inadequate food storage (failure to maintain the cold chain), the use of contaminated water and improper waste disposal in vending sites as recurring issues at vending sites (8–10). Tuglo et al. (8) reviewed 33 studies conducted in Ghana that included 6,095 food handlers between 2009 and 2022, and its findings revealed that only 55.8 percent of food handlers showed good food handling practices. Lack of food safety training was determined to be the most significant associated factor with an adjusted prevalence odds ratio (p = 0.001) of 0.10. A study by Desye et al. (11) based on 14 studies in low and middle-income countries confirmed that awareness does not necessarily translate to safe behavior, showing pooled proportions of 62 percent for good knowledge and 66 percent for positive attitudes amongst vendors, while good practices were found in only 51 percent.

Ghana has a developed legal system for food safety, based on the Public Health Act, 2012 (Act 851). Several agencies are involved in enforcement, such as the Food and Drugs Authority, the Ghana Standards Authority, and the Environmental Health and Sanitation Units of the district assemblies (12). However, the enforcement at the community level is still weak. According to Botha et al. (13), 40 food safety studies in Ghana reported that the regulatory bodies have generally failed to enforce food safety laws, conduct routine monitoring, and work effectively with each other. Inappropriate food safety practices by vendors in informal markets, the lack of trust in the regulatory system, and the lack of anonymity of vendors are the three key factors contributing to poor food safety practices in informal markets in Ghana (14). Poor hygiene practices by vendors are therefore not just the result of individual behavior but also the result of systemic institutional failures in the area of food safety governance.

Despite the size of the food vending business in socio-economically distinct communities (Asamankese, Adabraka, and Kpando Gabi), limited empirical studies have been found documenting vendor hygiene practices in any of these specific communities. The lack of evidence from these communities is part of a larger trend in the food safety literature to which this study directly contributes. Published food safety research in Ghana clusters in major urban centers such as Accra, Kumasi, and Tamale, while smaller towns all over the country are systematically missing from the published record (8, 13). Quantitative surveys tend to dominate the evidence that is available, and qualitative studies exploring the reasons for vendor behavior, how they experience regulatory enforcement, and what barriers they encounter in their practice day-to-day are limited (8, 14). The most widely cited qualitative study of street food safety behavior in Ghana, by Rheinlander et al. (15) in Kumasi, is now almost 20 years old, and no published study has since examined vendor hygiene qualitatively. Health authorities in these study communities have no locally grounded, qualitative information to inform training programs, inspection priorities, and public health communication, and this study was designed to fill that research gap.

This study, therefore, assessed the hygiene practices of food vendors in Asamankese, Adabraka, and Kpando Gabi. It specifically explored personal hygiene practices and behaviors of food vendors in these communities, and understanding the knowledge, attitudes, and beliefs of food vendors on food hygiene and food safety. The findings are significant for public health authorities, local government assemblies, and regulatory agencies in designing targeted hygiene education, improving inspection systems, and strengthening food safety enforcement. Additionally, the study highlights the need for practical infrastructure support and consistent regulation to reduce foodborne disease risks and protect consumers who rely heavily on street-vended foods.

## Methodolgy

### Research design

This study employed an ethnographic qualitative study design. This design allows for an in-depth examination of the lived experiences, behaviors, and perceptions of food vendors in their natural work environments as opposed to measuring or quantifying those behaviors through numerical indicators (16). Both Adaku et al. (14) and Rheinlander et al. (15) showed that qualitative approaches provide insights into the motivation of vendors and the experiences with regulations that structured surveys cannot access.

### Study areas

The selected study areas were Asamankese in the Eastern region, Adabraka in the Greater Accra region, and Gabo in the Volta region of Ghana. These sites were selected based on their unique socioeconomic and geo-demographic characteristics and spread across the country. The West Akim Municipal Assembly, with Asamankese as its capital, is one of the southern parts of Ghana's Eastern Region with an estimated surface area of 559 square kilometers. The 2021 Population and Housing Census shows the total municipal population as 120,145, comprising 48.5 percent males and 51.5 percent females (17). Asamankese lies about 75 kilometers northwest of Accra and is a commercial center with a fully developed traditional market that attracts traders from neighboring districts. The dominant economic activities include farming (44.6 percent), retail and wholesale trade (19.1 percent), and manufacturing (10.4 percent) (18). The most commonly reported diseases in the municipality are typhoid fever, diarrheal diseases, Cholera, several of which are directly related to failures in food and water safety. The health infrastructure of the municipality consists of one government hospital, one private hospital, five health centers, four clinics, and 32 Community Health Planning and Services compounds.

Adabraka is a well-established commercial neighborhood in Accra bounded from the north by Kawukudi and Alajo, from the east by Ring Road Central and Osu, from the south by the central business districts of Tudu and the Ministries, and from the west by Kojo Thompson Road and Darkuman. The area is dominated by a dense combination of residential housing, small businesses, local eateries, and open market stalls. The Adabraka Market, popularly known as the Saturday Market, is a market known for textiles, clothing, and other daily necessities. The Adabraka STC Station and the nearby Tudu Lorry park are key transit points that bring in high daily foot traffic during the working week. Prior field research in Accra found Adabraka as one of the neighborhoods in the capital that had the highest concentration of street food vendors (19).

Finally, Gabi is a town in the Kpando Municipality in the Volta Region, a few kilometers north of Lake Volta and near the larger townships of Kpando and Kpando Torkor. The Kpando Municipality is spread over 314.07 square kilometers, and the estimated population in 2017 is about 62,240, with 55.01 percent being categorized as urban (20). The local economy relies on agriculture, especially growing cassava, maize, yams, and vegetables, fishing, and small trading. The Kpando Market is the largest in the municipality, with a five-day cycle. Vendor screening is an identified public health priority in the municipal development plan. The North Dayi District, which borders Kpando Municipal to the south, was the site of a study by Tuglo et al. (21), which found that more than half of the food handlers had never attended any food safety training, thus providing the nearest published empirical benchmark of the Kpando Gabi setting.

### Study population and sampling

The study population consisted of all the street food vendors who were actively operating in the central business areas of the three townships at the point of data collection. Eligible Vendors include vendors of cooked meals, snacks, beverages, grilled foods, traditional food items, and fixed or mobile vending sites. Inclusion criteria included participants who were 18 years or older, directly involved in the preparation or sale of food, and gave informed consent to participate. Vendors not directly involved in food preparation or food sales and persons not willing to provide consent were excluded from the study.

Convenience sampling was used to select study participants. This research method relies on selecting individuals based on their availability and accessibility at the time of data collection (16). This method was also used because vendors were available mainly during business hours and enabled researchers to efficiently gather information within limited time constraints. Participants were recruited to be part of the study by being present at the vending site at the time of the research visit, meeting the inclusion criteria, and providing consent to participate. The final number of participants was 20 based on data saturation, that is, a point where new interviews no longer provide new codes or add new thematic content (22).

### Data collection

Structured field observations were conducted at 20 food vending locations at the three locations. Each site was observed for about 30 to 60 min during busy business hours to observe routine practices of vendors under normal operating conditions. Observations include personal hygiene behaviors such as handwashing practices, use of Personal Protective Equipment (PPE), food handling and storage practices, waste disposal, environmental cleanliness, and management of interactions between food, money, and customers. A standardized observation checklist was used in the same way at all of the sites to ensure systematic and comparable data collection across the research team. Field notes recording contextual details, behaviors not included in the checklist, along with the physical conditions of each site, are recorded immediately after each observation session.

Structured in-depth interviews were held with all consenting participants. Each interview was done by visiting the participant's home or place of work at a time convenient to the participant, and takes about 15 to 25 min to book ahead for the main interview. The interview guide addressed five thematic areas, which are background information and training history, personal hygiene routines before and during work, environmental and food handling practices, knowledge and attitudes about food hygiene and foodborne disease, and experiences with regulatory inspections and enforcement. The guide contains open-ended questions that encourage participants to describe their experiences and practices using their own words, as well as more specific questions on behaviors that have been identified in the preceding literature as an area of concern. Interviews were conducted in English or in the local language, as appropriate, and audio recorded with the participant's consent. All of the recordings were transcribed after each interview.

Participants were interviewed face-to-face at the vending sites during operating hours. Vendors who did not meet the inclusion criteria or who refused to participate at the time of approach were not enrolled; the most frequently cited reason for non-participation was being too busy to speak during peak trading hours. No repeat interviews were conducted, as each interview generated sufficiently rich and detailed information to address the study objectives, and thematic saturation was achieved through the single in-depth interviews. Additionally, the nature of the vendors' work and their limited availability during business hours made scheduling follow-up interviews impractical. During field observations, customers and passers-by were sometimes present at the vending site, although no third party was present during any individual interview. The interview guide was pre-tested with two food vendors outside the study sites prior to the main data collection phase to ensure clarity and appropriateness of the questions, and minor revisions to the wording were made based on the pre-test findings.

### Data analysis

The transcribed data was analyzed using reflexive thematic analysis as described by Braun and Clarke (22, 23). The analytical process went through six phases. In the first phase, the entire dataset of interview transcripts and field notes was read repeatedly to gain familiarity with the data. In the second phase, initial codes was generated systematically across the dataset by each of the research teams. In the third phase, related codes was grouped into candidate themes. In the fourth phase, the candidate themes were reviewed against both the full coded dataset and the original transcripts for coherence, specificity and coverage. In the fifth phase, final themes were named and clearly defined. In the sixth phase, the analysis was written up as an account that makes direct reference to the three main research questions. Field notes were also included with the interview data to add observational triangulation. Member checking was done by presenting preliminary themes back to a sample of the participants to enhance the credibility of the analysis.

Codes were directly derived from the data and organized under the three research questions that guided the study. Transcripts were not returned to participants for correction as the process of member-checking with preliminary themes were the mechanism for participant feedback on the analytical output.

### Reflexivity and research team

The interviews and field observations were carried out by trained research assistants. The trained on qualitative interview technique and structured observation before data collection began.

### Ethical considerations

Ethical clearance was obtained from the relevant institutional review board of the University of Health and Allied Sciences in accordance with the Helsinki declaration. Informed consent was sought from every participant before any interview or observation took place. Participants received a clear explanation of the study's purpose, the methods involved, the expected duration of their participation, and their right to withdraw at any time without consequence. All recordings, transcripts, and observation notes were stored securely on password-protected devices and assigned participant codes to protect confidentiality. No identifying information appears in any research output, conference presentation, or publication arising from this study.

## Results

**FIGURE 1:**
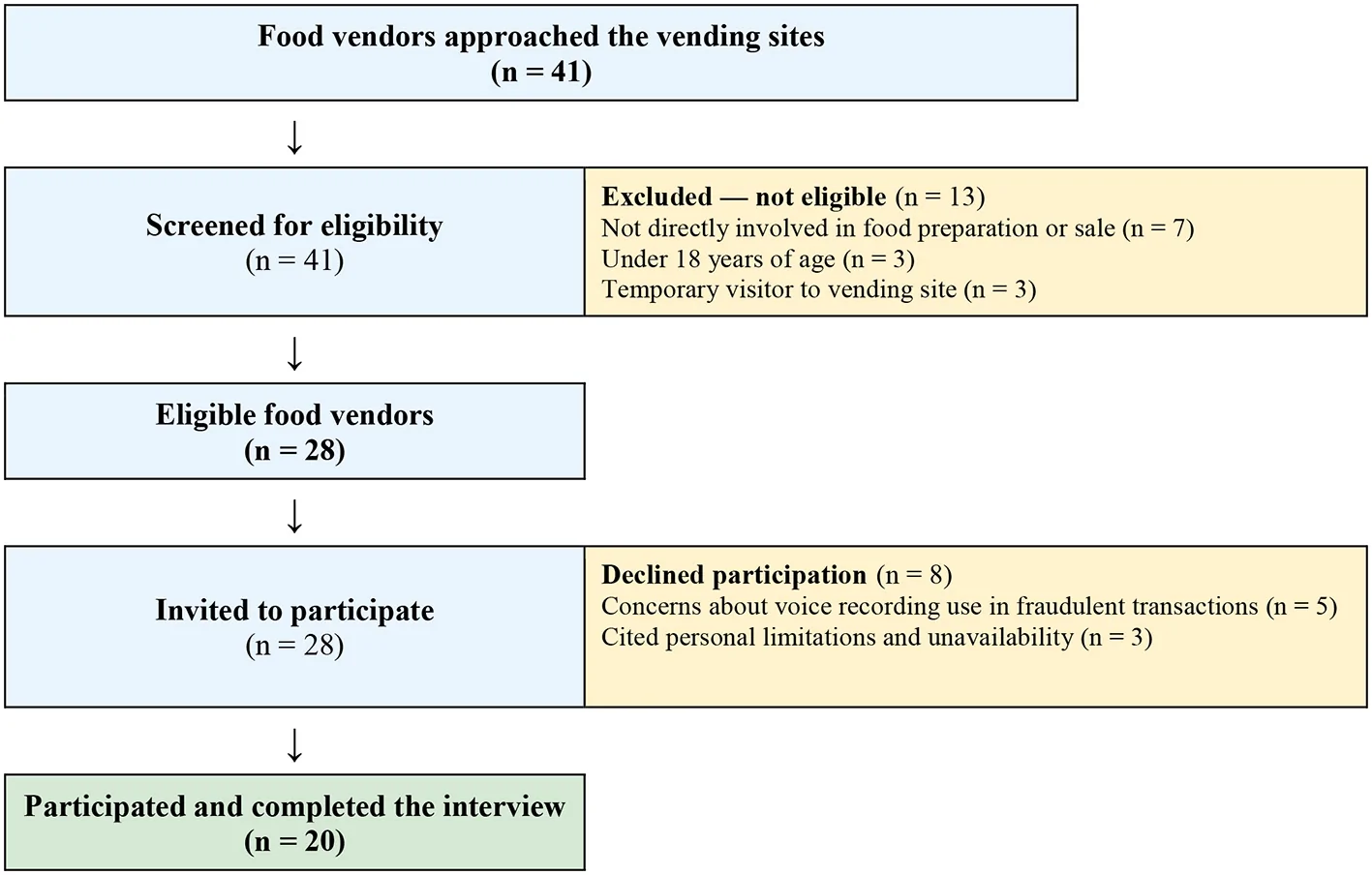
Flowchart illustrating the participant recruitment and selection process for the ethnographic qualitative study on hygiene practices among food vendors in Asamankese, Adabraka, and Kpando Gabi (n = 20).

A total of 41 food vendors were approached to participate in the study at the three study sites ie. Adabraka, Kpando and Asamankese. After following the inclusion criteria, 13 were deemed ineligible and excluded. Reasons for ineligibility included not being directly involved with food preparation or sale, being under 18 years of age, and being a temporary visitor to a vending site instead of being a resident vendor; this resulted in 28 eligible vendors, all of whom were formally invited to participate Following this process, eight refused to participate. The main reason given for declining was concern about how voice recordings might be used in fraudulent transactions and other situations not related to the study, personal limitations and unavailability at the time of the study. The remaining 20 vendors agreed and completed the structured in-depth interview. Figure 1 summarizes the flow of participant selection from the pool of approached vendors to the final sample.

### Socio-demographic characteristics of the participants

The socio-demographic characteristics of participants are summarized in Table 1. Eighteen (90.0%) of the 20 participants were female, and two (10.0%) were male. Eight of the participants (40.0%) were from Adabraka, seven (35.0%) from Kpando Gabi, and five (25.0%) from Asamankese. In terms of years of vending experience, 4 (20.0%) had been in the food vending business for less than 5 years, 5 (25.0%) between 5 and 10 years, and 4 (20.0%) reported more than 20 years. For five participants (25.0%), years of experience were not reported. Most participants sold cooked local meals, including kenkey, banku, fufu, rice and soup, representing 15 (75.0%) of the sample. Four (20.0%) sold light food and snacks. Formal food safety training by attending a catering school was reported by only four participants (20.0%), and 16 (80.0%) had no such formal training. Most of these vendors had learned food preparation from a family member, a previous employer, or by teaching themselves.

**Table 1:** Socio-demographic characteristics of the participants.

Variable	Frequency (*n* = 20)	Percentage (%)
Sex		
Male	2	10.0
Female	18	90.0
Study site		
Adabraka	8	40.0
Kpando Gabi	7	35.0
Asamankese	5	25.0
Years of vending experience		
Less than 5 years	4	20.0
5 to 10 years	5	25.0
11 to 20 years	2	10.0
Over 20 years	4	20.0
Not stated	5	25.0
Type of food sold		
Cooked local meals (Kenkey, banku, fufu, rice, soup)	15	75.0
Light food and snacks (tea, bread, salad, eggs)	4	20.0
Not specified	1	5.0
Formal food safety training (catering school)		
Yes	4	20.0
No	16	80.0

### Thematic findings

**Figure 2:**
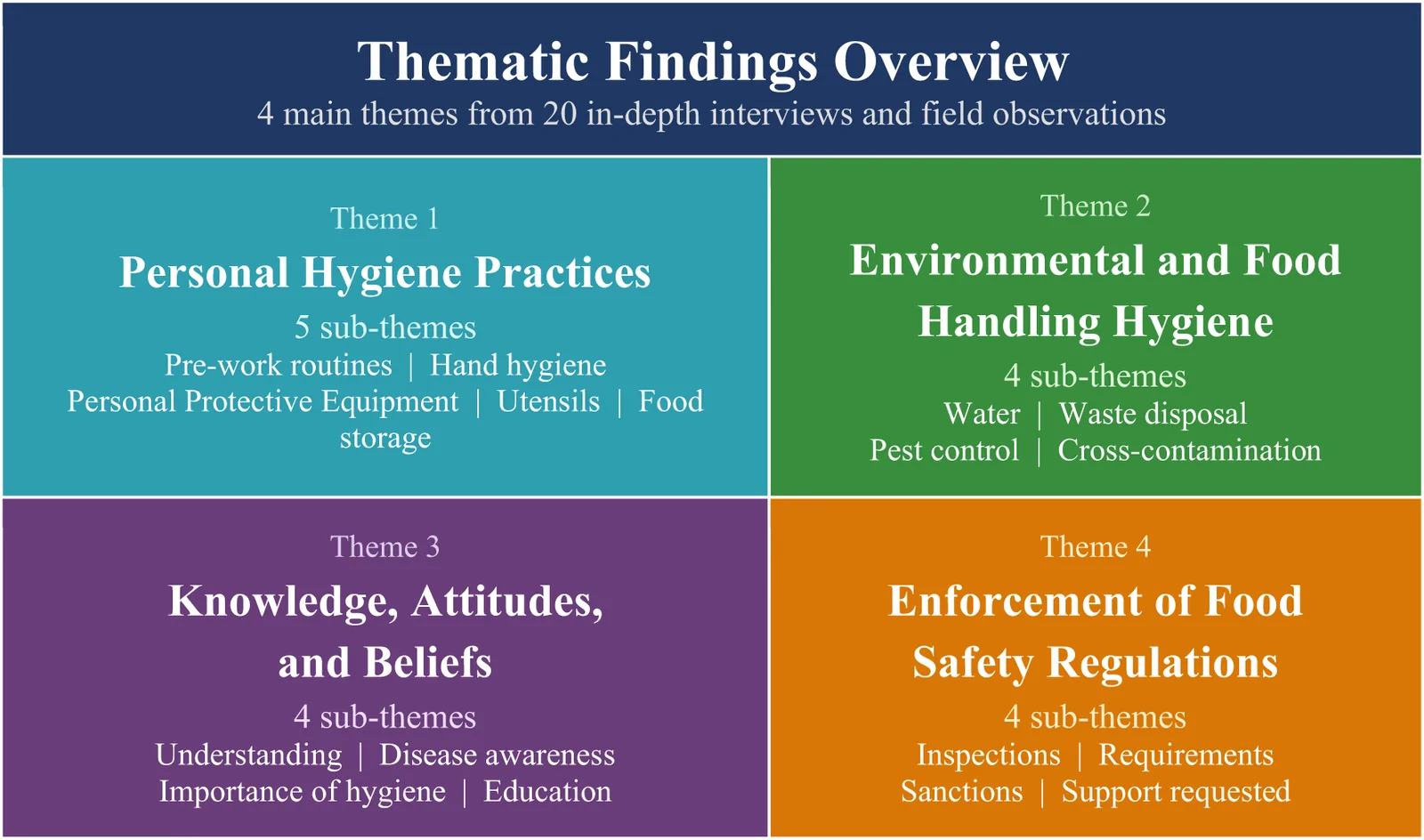
Four main themes identified from structured in-depth interviews and field observations with 20 food vendors in Asamankese, Adabraka, and Kpando Gabi.

Table 2 presents the themes of hygiene practices among food vendors for analysis. Major themes included personal hygiene measures, environmental and food handling hygiene, knowledge, attitudes, and beliefs regarding food hygiene, and enforcement of food safety regulations. Figure 2 gives a pictorial view of the key thematic areas and their sub-themes.

**Table 2:** Thematic table.

Main Theme	Sub-theme	Codes
Personal hygiene practices	Pre-work hygiene routines	Bathing before work, sweeping and cleaning the vending site; Washing utensils before starting; Wearing clean clothing.
	Hand hygiene during food handling	Regular handwashing with soap; Handwashing between tasks; Use of rubber gloves; Use of hand sanitiser; Bowl of water for handwashing; Same hand used for food and money
	Use of Personal Protective Equipment	Head cover to prevent hair in food; Apron use; Rubber gloves; Nose mask; Removal of protective items due to heat.
	Utensil cleanliness	Washing utensils after each use; Washing at close of day; Drying in the sun; Covering stored utensils
	Food storage practices	Refrigerator for cooked food or proteins; Ice chest use; Reheating leftovers; Daily fresh purchase; No leftovers at close
Environmental and food handling hygiene	Water source for cooking and cleaning	Pipe water (Ghana Water Company); Rainwater; Storage in a tank or covered container; Boiling before use
	Waste disposal	Covered dustbin; Waste collected by Zoom Lion or AMA; Tying waste in rubber bags; Burning of waste; Giving leftover food to animals
	Environmental pest and dust control	Food net covers; Mosquito coil; Insecticide spray; Covering food when dust is present; Gating the vending area; Road dust as a recurring challenge
	Prevention of cross-contamination	Separating raw and cooked food; Covered containers; Refrigerating raw protein; Inspecting food for spoilage before use; Daily fresh purchases; No clear method.
Knowledge, Attitudes, and Beliefs about Food Hygiene	Understanding of food hygiene	Preventing contamination and disease; Keeping the environment clean; Personal cleanliness; Safe food preparation and handling.
	Perceived diseases from poor hygiene	Cholera; Typhoid; Diarrhea; Food poisoning; Stomach problems; Ulcer; Malaria
	Perceived importance of food hygiene	Attracts customers; Prevents illness in the vendor and customers; Protects family; Business motivation; Personal health motivation
	Receipt of food hygiene education from health officers	Clinic or health worker visits; Market-based group education; Blood testing and card renewal; No education received
Enforcement of food safety regulations	Inspection experiences	Annual or twice-yearly blood testing; Monthly hygiene checks; Infrequent or no inspections received; Issuance of health cards
	Regulatory requirements for vending	Food vendor health card; Blood test for disease clearance; Registered place of work; Hygiene equipment (gloves, sanitiser, nets); Council examination
	Sanctions and warnings	Verbal instructions to maintain cleanliness; Temporary suspension of operations; Warning about vending location; Instruction to clean gutters; No sanctions received
	Support is requested from the authorities.	More frequent training and education; Provision of dustbins; Supply of gloves, sanitisers, and food covers; Stricter and consistent enforcement; Drainage system improvements; Routine health checks

### Personal hygiene practices

**Figure 3:**
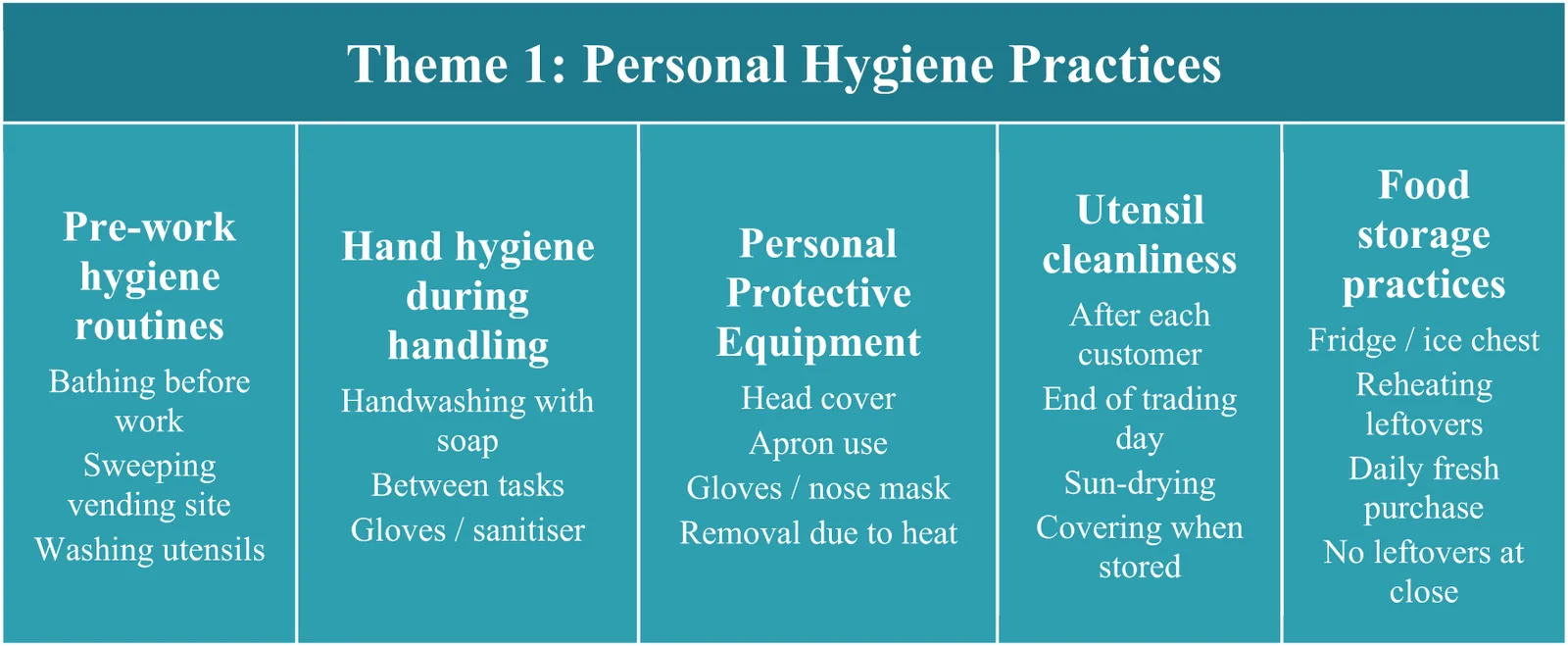
Five sub-themes under personal hygiene practices, with representative codes for each sub-theme.

The results identified five sub-themes, including pre-work hygiene routines, hand hygiene behavior during food handling, Personal Protective Equipment usage, cleanliness of utensils and how food is stored (Figure 3). These sub-themes give an in-depth account of the personal hygiene behaviors that vendors adopted before and during food preparation and service. Most vendors described a morning routine of bathing, sweeping the vending site and washing utensils before cooking, although the thoroughness of these routines varied between sites and individuals.

Regarding pre-work hygiene routines, most vendors across all three sites described bathing, sweeping the vending area, and washing utensils with water and detergent before commencing work. These actions were interpreted as basic requirements before serving customers. Participants reported that,

“*When I wake up, I brush my teeth and take my bath, after my kid go to school, I start cooking.” (Female, Kpando Gabi)*

“*I come to my food joint at 5 am in the morning. I clean the place. I wash the bowl that I use to serve the customers. I fetch water. I clean my shoes, dustbins and a lot of things.” (Female, Adabraka)*

Concerning hand hygiene during food handling, all participants said that they knew that they had to wash their hands before and during food handling. However, field observations at several sites consistently indicated that vendors did not wash their hands during the observation period and most handled food and money with the same hand. This was a pattern observed at sites in all three communities, suggesting a difference between what was described by vendors and what was seen. Participants stated that,

“*We wash our hands before starting everything. While working and then you get up to do something, you have to wash your hands before you continue again.” (Male, Adabraka)*

“*I wash my hands with soap and water neatly before I start, even after I bath I wash my hands before I start.” (Female, Kpando Gabi)*

The fact that most vendors did not wash their hands between serving food and collecting money, even though they reported doing so, indicates a behavioral disconnect that awareness alone has not addressed.

With respect to the use of Personal Protective Equipment, the head covering to prevent hair from falling into food was the most consistent practice described by participants at all three sites. The use of aprons and rubber gloves was also mentioned, though inconsistently. Removal of head covers and aprons during the working day because of warmth was reported at all sites, but field observations indicated that protective items were missing at several vending sites at the time of visit (see Field Observation Findings). Participants reported that,

“*After bathing, I wear a long dress and cover my head, as for me I always cover my head because it prevents hair from entering the food. Most at time when customers see hair in the kenkey, it stops them from buying next time.” (Female, Kpando Gabi)*

“*As a professional, you need to cover your head, because your hair, sometimes it can drop into the food, so you need to cover them. It's part of the hygiene. Your nails must be short all the time. And your apron must be neat all the time.” (Male, Adabraka)*

In terms of the cleanliness of utensils, all participants indicated that they clean utensils, but at different times (or frequency). Some were washed at the end of each customer, others at the end of the trading day. Drying utensils in the sun and covering them when stored were also mentioned. Participants stated that,

“*After they finish eating, immediately they finish eating, I take it, I wash it, I place it in the basket and I cover it.” (Female, Adabraka)*

“*After cooking we pour water into the banku pot to soften it so we wash.” (Female, Kpando Gabi)*

Concerning food storage practices, methods for the handling of leftover food and ingredients varied mainly depending on access to refrigeration. Vendors in Adabraka more often had access to fridges or freezers compared to those in Kpando Gabi and Asamankese, where reheating and ice chests were used. Fresh daily purchases were also reported by some Adabraka vendors, which minimized the amount of food that had to be stored overnight. Participants reported that,

“*I heat the leftover foods, because I do not have fridge.” (Female, Kpando Gabi)*

“*Leftovers, for the veggies, I store them in the fridge. For the green groceries, you have to put them in the fridge, in order not to rot. Because you don't have to use rot items to prepare food for people.” (Male, Adabraka)*

### Environmental and food handling hygiene

**Figure 4:**
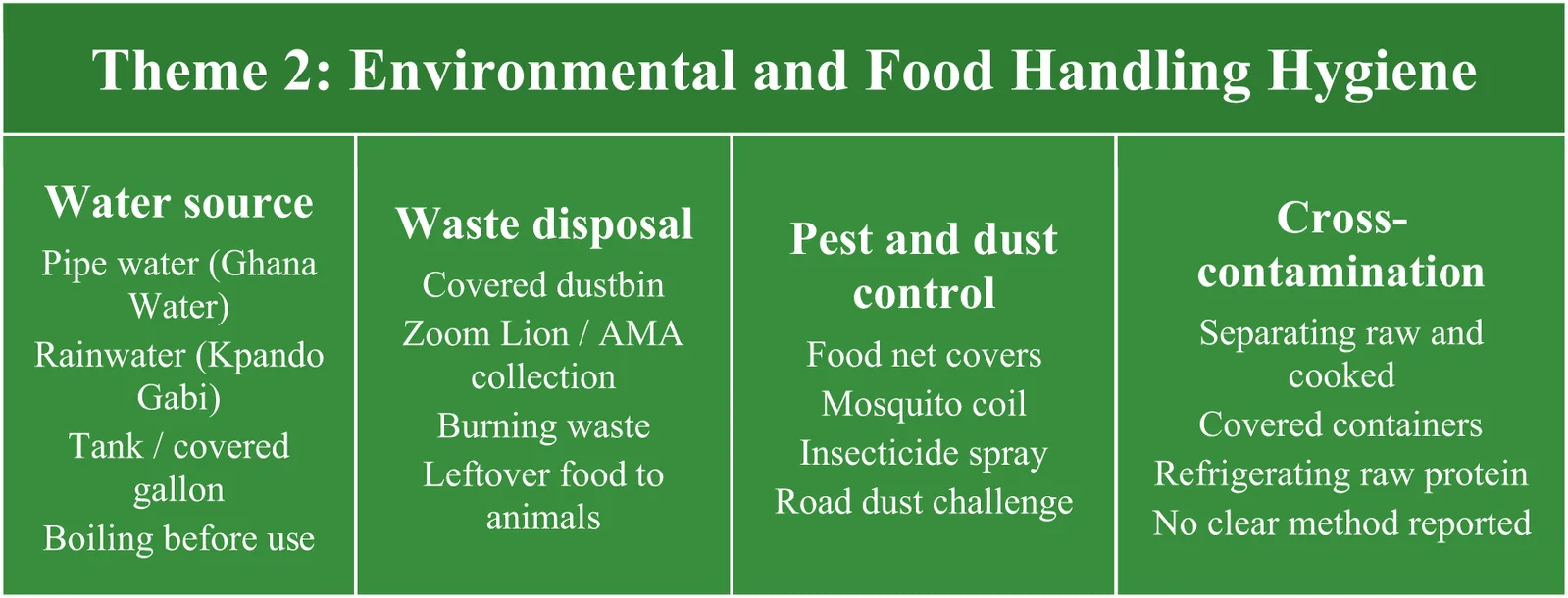
Four sub-themes under environmental and food handling hygiene, with representative codes for each sub-theme.

Under this main theme, four sub-themes were discovered: water source for cooking and cleaning, waste disposal, environmental pest and dust control, and prevention of cross-contamination (Figure 4). These sub-themes describe how vendors managed the physical environment surrounding their vending sites and managed food as it relates to contamination risks.

The most reported main water source used for cooking and cleaning is piped water from the Ghana Water Company in all three sites. Most vendors stored water in tanks or covered gallons and believed the water was safe to use without additional treatment. A few vendors from Kpando Gabi depended on rainwater, and sometimes this was the only water source available. Participants stated that,

“*We have tap water, Ghana water. It's mostly in a tank and then in gallon, well covered.” (Male, Adabraka)*

“*Pipe water, we fetch it.” (Female, Kpando Gabi)*

Concerning waste disposal, the majority of vendors in Adabraka used covered dustbins, and waste was collected by Zoom Lion or the Accra Metropolitan Assembly. In Kpando Gabi, the opportunities for formal waste collection were fewer, and some vendors burned waste or provided food left by customers to animals. This disparity shows differences in infrastructure between sites that determined waste management options. Participants reported that,

“*With dustbin. Like the zoom lion comes picking up in the morning. We just pack it in the dustbin.” (Female, Adabraka)*

“*I burn the rubber and gather the leftover food from customers to pigs.” (Female, Kpando Gabi)*

Field observations at one Kpando Gabi site recorded an uncovered refuse bin situated near the food preparation area, a condition that raises the risk of food contamination from pests and pathogens (see Appendix A.1, Photo Gallery).

With respect to environmental pests, vectors, and other hazards, the most commonly reported hazards in all three sites were flies and road dust. Food net covers were most widely used to manage flies and were supplemented by mosquito coils and insecticide sprays. Road dust from passing vehicles was described as a constant problem for roadside vendors, especially in Adabraka. Rodents, vermin, and furry animals were also reported as pests at sites within all three communities. Participants reported that,

“*Dust, yes, because the road is not tight. So we always encounter it. But we use food covers for the serving bowls. And we cover our food very tightly.” (Male, Adabraka)*

“*When I sell the food, and I see flies, I just put on this thing, mosquito pills. It sends them away. And when I close, I spray the whole place. So, the moment I come in the morning, they will all die.” (Female, Adabraka)*

With regard to preventing cross-contamination, there were uneven practices for keeping raw and cooked food separate among the participants. Some Adabraka vendors stored the raw proteins in a fridge or covered container separated from the cooked food. Others, especially in Kpando Gabi and Asamankese, were unable to say any specific method, and one Adabraka vendor said she had no idea how to avoid cross-contamination. These results indicate that cross-contamination control was the least understood hygiene practice in this sample. Participants characterized their approaches as follows,

“*I only store raw food in the freezer. I don't normally store cooked foods in the freezer. But only the raw ones in the freezer.” (Female, Adabraka)*

“*After grinding my maize, I put it in a covered container, also with tomato, I blend it with salt for preservation because I do not have a fridge, with fish, I buy the already fried one.” (Female, Kpando Gabi)*

### Knowledge, attitudes, and beliefs about food hygiene

**Figure 5:**
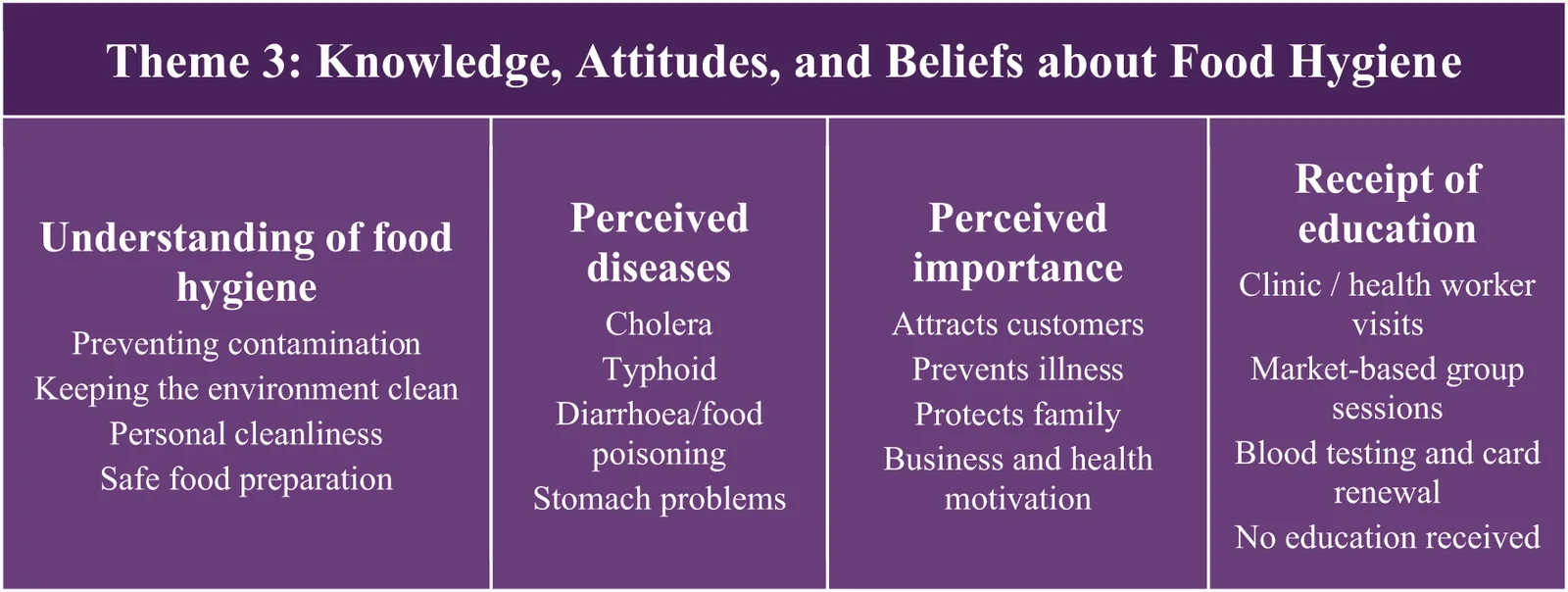
Four sub-themes under environmental and food handling hygiene, with representative codes for each sub-theme.

Under this theme, 4 sub-themes emerged: understanding of food hygiene, perceived diseases from poor hygiene, perceived importance of food hygiene, and receipt of food hygiene education from health officers (Figure 5). These sub-themes capture how vendors understood, valued, and received information on food hygiene.

Regarding the understanding of food hygiene, most of the participants described food hygiene in a practical, everyday language. The most common descriptions were around keeping the environment clean, personal cleanliness, and preventing illness in customers. A small number failed to provide any definition when the term was introduced in the interview; others defined it in terms of its outcome rather than its definition. Participants stated that,

“*Food hygiene, per se, is a way of keeping things neat in order not to infect or give diseases to people.” (Male, Adabraka)*

“*Cooking in a clean environment and practicing personal hygiene.” (Female, Kpando Gabi)*.

Concerning perceived diseases from poor hygiene, cholera and typhoid were the most frequently mentioned diseases at all three sites. Diarrhea, food poisoning, stomach problems, and ulcers were also cited. One vendor uses the name malaria, which represents a misattribution that may need to be addressed during training. The identification of flies as the vehicle for typhoid transmission occurred in accounts from all three sites and represents a functional, if informal, understanding of the pathways of contamination. Illustrative quotes included,

“*Cholera. It comes from poor hygiene. Not handling your food, water, and other things well.” (Female, Adabraka)*

“*Firstly, typhoid is the number one thing. If you are not careful, it's not easy to treat it. So when you don't handle things well, especially from the flies and those things, and they contaminate people, that means you are spreading the disease to everybody.” (Male, Adabraka)*

With regard to the perceived importance of food hygiene, the participant responses were all in agreement that food hygiene was important. The two most common reasons were customer attraction and personal and family health protection, which were frequently stated jointly. This indicates that hygiene was perceived as both a business need and a health issue at the same time. Participants reported that,

“*People come from afar to buy from me because my food is good and hygienic, it helps me get my daily bread every day.” (Female, Kpando Gabi)*

“*It's the number one key. When you do anything anyhow and give it to 30 people a day, and it's not the right thing, you have infected the 30 people.” (Male, Adabraka)*

In terms of the receipt of food hygiene education from health officers, vendor contact with health authorities varied between sites. Vendors located in Adabraka reported more frequent contact, group activities in the market, and visits to the site. Health workers sometimes visited to make observations and provide corrections in Kpando Gabi. In Asamankese, blood testing and card renewal were major activities related to visits to health authorities. Several participants across the three sites reported that they had never received food hygiene education from a health officer. Participants reported that,

“*They come from the clinic to teach us not to allow flies around the food, how to wash our hands, and cooking in a hygienic environment.” (Female, Kpando Gabi)*

“*The nurses call on us. They do public environmental health. They call us, and they give us training about how we take care of the food that we sell.” (Female, Adabraka)*.

### Enforcement of food safety regulations

**Figure 6:**
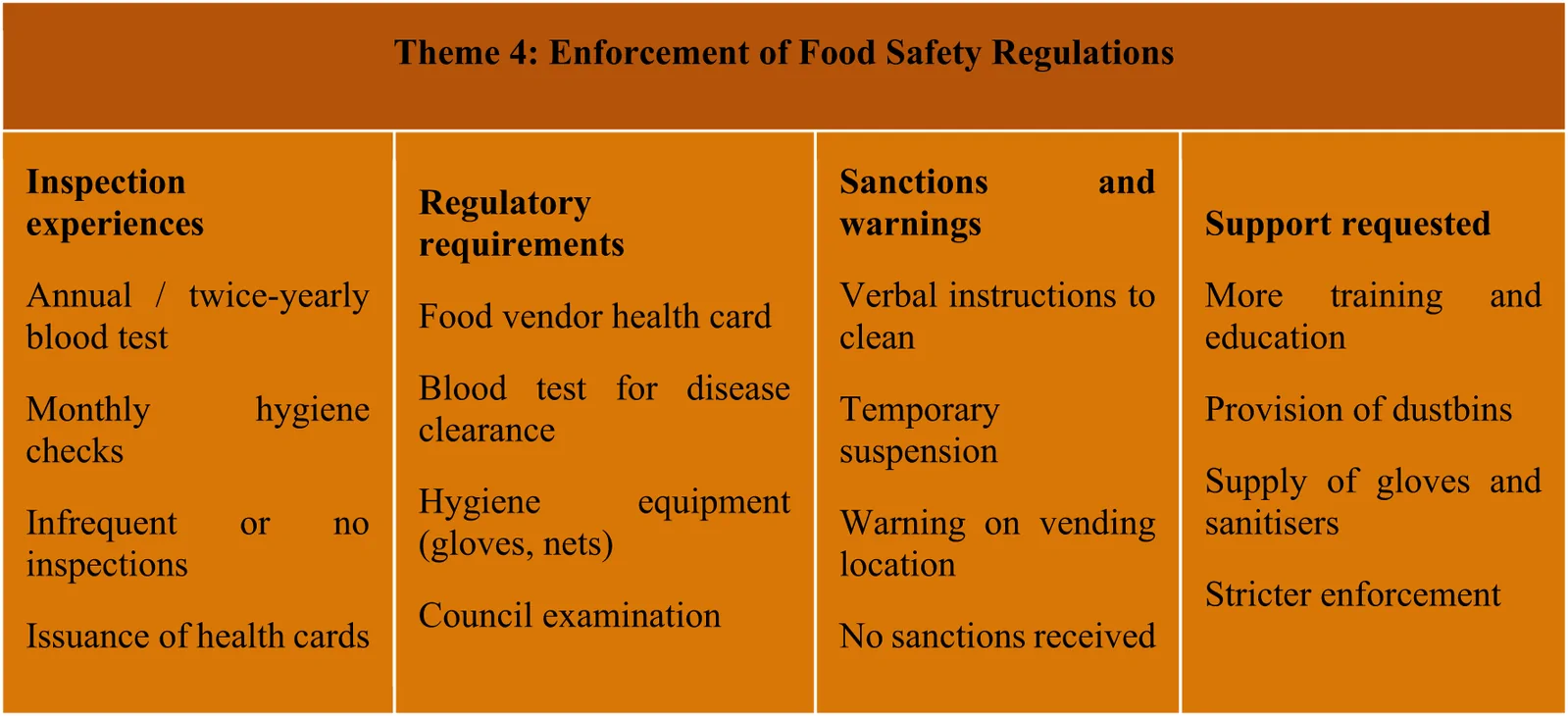
Four sub-themes under enforcement of food safety regulations, with representative codes for each sub-theme.

Under the theme, four sub-themes were identified: inspection experiences, regulatory requirements for vending, sanctions and warnings, and support requested from authorities (Figure 6). These sub-themes describe the nature and frequency of regulatory contact experienced by vendors, what was asked of them, and what they sought.

With respect to experiences during inspections, the contact of vendors with health authority inspectors varied considerably. Some Adabraka vendors described monthly hygiene checks and annual blood testing, while others across all three sites described only one inspection visit - over several years, or none at all. The content of inspections, where they took place, was more about blood tests and health card issuance than the physical conditions of the vending site. Participants reported that,

“*They are going to check your blood and everything to see if there's any disease, so that you get treated, because you can't be sick and deliver food to people.” (Female, Adabraka)*

“*One came at home, and he even took a picture of us. That is the last two years or three years or so. Since then, no one came.” (Female, Adabraka)*

With regards to regulatory requirements on vending, the most frequently mentioned requirement across sites was a food vendor health card obtained following blood testing. In some cases, vendors were also asked to procure basic hygiene equipment like gloves, sanitiser, and food net covers. However, several vendors said that no requirements had been communicated to them before they began selling. This could imply that regulatory entry requirements are not always applied or enforced in an equal way across communities. Some participants said that,

“*They said that I should get a card. That shows that I don't have any disease. Second, they asked me to get a net and also various liquor buckets, gloves, liquid soap, sanitisers, and a lot of things.” (Female, Adabraka)*

“*We take cards from the council and write exams, they teach us how to treat ourselves when sick.” (Female, Kpando Gabi)*

With regard to sanctions and warnings, the majority of participants indicated that they are not formally sanctioned. Where warnings had been made, these were verbal instructions to make the site tidier or to correct a specific situation. One vendor reported a temporary suspension of operations of about two weeks. Vendors raised a concern that inspectors, in some instances, negotiated with non-compliant vendors rather than enforcing rules. Some participants said that,

“*They complain about the place I sell my food, but this is the only place I have. I have been here for over 16 years.” (Female, Asamankese)*

“*A lot of times they sanction us to clean our gutters and environment.” (Female, Asamankese)*

“*They should give us a dustbin. They should get a place for a dustbin for us to be. After work, it should be easy for you to dispose of your things inside. And then they should also be educating us on how to go about food hygiene.” (Female, Adabraka)*

“*They can come around and organize a programme for us street sellers so they can educate us on certain things so that we get to know more, because most of us selling food like this, we are sometimes not educated, we just wake up and start our business.” (Female, Adabraka)*

### Field observation findings

Structured observations were undertaken to triangulate the interview findings and to compare vendors' reported food hygiene practices with actual practices observed at the vending sites. Observations were conducted at all 20 vending sites across the three communities for 30–60 min during peak trading hours. The observations focused on hand hygiene, use of Personal Protective Equipment, water storage, utensil management, waste disposal, and environmental sanitation. Selected photographic evidence is presented in Appendix A.1.

Hand hygiene was the most consistent discrepancy between what vendors reported and what was found. No vendor was observed to wash their hands between handling food and collecting money during any observation period from all three sites. At one or two of the sites, there was a bowl of water available near the food preparation area, but it was not used during the observation window. At one Adabraka site, the same bowl of water was used by several different vendors for different tasks without being replaced.

Protective clothing was missing or incomplete at most sites where it was present. Head covers were not observed at some of the sites in Kpando Gabi and Asamankese during the observation period. Aprons were observed in only a few Adabraka sites. No gloves were used during food service at any of the 20 sites. At sites where there were protective items, they were frequently set aside when actively handling food or serving customers.

Conditions of water storage differed between locations. Large uncovered drums and open basins were the most common water storage containers found in Kpando Gabi and Asamankese sites (Appendix A.1). In Adabraka, covered gallons and fitted dispensers were more prevalent, as were improvised stands and placement on the floor. No site in Kpando Gabi or Asamankese was seen to treat stored water before use.

Utensil management was erratic. At several sites, washed bowls and plates were against open shelves or placed on a wooden surface and were exposed to dust, flies, and other contaminants (Appendix A.1). At one Kpando Gabi site, used bowls were stacked directly on a dirty floor surface pending washing. In Adabraka, some of the vendors had clean utensils in covered baskets, but this was certainly not the norm across the sample.

Environmental circumstances at several sites posed risks of food contamination. An uncovered refuse bowl with food waste was found next to the cooking area at one Kpando Gabi site (Appendix A.1). Wet and dirty floor surfaces had been documented at one of the Adabraka market stalls, where standing water was present near food storage shelves. Flies were observed landing on uncovered food or serving bowls in three locations in the three communities during the observation period. The physical conditions recorded from site to site are consistent with the interview accounts and suggest that the disparity between reported and practiced hygiene is evident in the vending environment itself.

## Discussion

This study explored hygiene practices, knowledge, attitudes, and regulatory experiences of food vendors in Asamankese, Adabraka, and Kpando Gabi using structured in-depth interviews and observations in the field. The results showed that most vendors were aware of food hygiene and could recognize foodborne diseases, but there were observable gaps in handwashing, the use of Personal Protective Equipment and the prevention of cross-contamination at all three sites. Environmental conditions such as waste management, pest control, and access to water varied depending on available infrastructure. Health authority inspections were sporadic and mainly involved blood testing and health card provision, and regulatory entry requirements were not consistently communicated to vendors.

The poor practice of hand hygiene in this study is a direct public health concern. Although all participants reported washing hands before and during food handling, field observations revealed that handwashing was not practiced during serving at most sites, and most vendors handled food and collected money with the same hand. This gap between stated and observed practice is consistent with the findings of Desye et al. (11), whose meta-analysis of 14 studies in low and middle-income countries found that only 51% of food vendors demonstrated good practices even though 62% reported good knowledge. In line with this, Tuglo et al. (8), in a systematic review and meta-analysis of 33 studies conducted in Ghana, found that only 55.8% of food handlers demonstrated good food handling practices, with lack of food safety training identified as the most strongly associated factor with an adjusted prevalence odds ratio of 0.10. These findings collectively suggest that awareness of the hygiene requirements does not necessarily transfer automatically into safe behavior, and that without practical and site-based training, the difference between the reported and the observed practice will remain.

The inconsistent use of protective clothing, especially the removal of head covers and aprons during the working day on account of heat, was another practice observed at all three sites. Addo-Tham et al. (9) also reported a similar level of irregularity in the use of protective items among food vendors in the Ejisu-Juaben Municipality of Ghana, where vendors recognized the use of protective gear but attributed different reasons, such as discomfort for not wearing them regularly. Nkosi and Tabit (10) found the same trend among street food vendors in the Zululand District of South Africa, with head covers and aprons being the most commonly set aside during service. These findings in various settings would indicate a common obstacle, which is that the design and wearability of protective items in hot and open environments are not adequately addressed in standard food hygiene guidance.

The food storage practices in this study were determined by access to electricity and refrigeration, not vendor knowledge. Vendors in Adabraka, the most urban of the three sites, had more access to fridges and freezers, whereas vendors in Kpando Gabi relied on reheating and ice chests. Reheating leftovers, if it is not reheated to an adequate temperature, does not kill pathogens that may have multiplied during storage. This disparity in the amount of storage between urban and rural vending environments is similar to the overall trend reported by Salamandane et al. (2) in their systematic review of street food vending in developing countries, which indicated that infrastructure access, such as electricity and refrigeration, is a repeated equation for food safety outcomes in informal food environments in sub-Saharan Africa.

Concerning environmental and food handling hygiene, pipe water from the Ghana Water Company was the main source of cooking and cleaning water for most of the vendors, consistent with findings from urban and peri-urban settings documented by Tuglo et al. (8). However, the use of untreated rainwater by some of the Kpando Gabi vendors represents a contamination risk that is not covered by pipe-water-based guidance. Botha et al. (13), in a scoping review of 40 food safety studies in Ghana, identified that inconsistent and unreliable water supply was a recurring food safety concern in urban and rural settings in the country. Waste disposal practices varied greatly from site to site, with burning and informal disposal of waste reportedly in Kpando Gabi, in contrast to formal collection services in Adabraka. These disparities do not indicate variations in the intent of vendors, but in unequal municipal infrastructure, a distinction that should be considered in intervention design. The presence of rodents, flies, dust, and animals at vending places in all three communities is aligned with the conditions reported by Nicolo and Ferroni (19) in Accra, where exposure to pests at the open-air vending places was a constant, structurally induced issue.

Cross-contamination prevention was the poorest area of practice for the sample. Several vendors, especially in Kpando Gabi and Asamankese, were unable to explain any method for separating the raw and cooked food. One Adabraka vendor explicitly said she had no idea how to tackle it. Desye et al. (11) in their meta-analysis found that knowledge of cross-contamination control was one of the least common specific food safety competencies, with 51% of vendors in low and middle-income settings demonstrating correct knowledge/skills. The lack of knowledge of this is of particular concern since cross-contamination of raw proteins and ready-to-eat foods is a direct route of transmission for pathogens which cause cholera, typhoid, and diarrhoeal disease, the same diseases most frequently listed by participants as a consequence of poor hygiene.

The field observations and photographic documentation in Appendix A.1 support some of the interview findings. Images taken at vending sites in Kpando Gabi and Asamankese indicate water being stored in large open drums and basins that were not covered - consistent with the interview accounts of storing water from pipes in tanks and gallons. At the same sites, washed utensils were photographed while they were stored in the open on wooden surfaces or on shelves without any kinds of covers, leaving them exposed to flies, dust, and other environmental contaminants. One of the images (shown from K pando Gabi) is a refuse bowl with food waste sitting next to a food preparation surface, which is a direct reflection of the field observation note about the uncovered refuse bin described under Environmental and Food Handling Hygiene. A photograph taken at an Adabraka location depicts a wet and dirty floor surface around food storage shelves, which confirms the reports of inadequate drainage and environmental maintenance at the same site. These photographs support the finding that the gap between reported hygiene practice and actual vending conditions is a physical and observable one, and not just a matter of self-report.

Most of the vendors in this study explained food hygiene in practical and informal terms, focused on cleanliness of the environment and personal neatness rather than microbial contamination. This is reflected by the finding of Rheinlander et al. (15), who conducted a qualitative study of street food vendors in Kumasi, Ghana, and found that vendors understood hygiene in terms of appearance and customer perception. In line with the current study, Rheinlander et al. (15) found that the prevention of visible uncleanliness, rather than unseen microbial risk, was the dominant frame through which vendors evaluated their own practice. Customer attraction was the most common reason for maintaining hygiene, followed by personal and family health. Isanovic et al. (24), in a six-country study in Africa and Asia, found that vendor behavior was highly influenced by customer perception and social reputation, and that this external accountability mechanism often influenced vendor behavior more than health knowledge. This study confirms that finding. However, a vendor motivation based on customer perception, rather than disease prevention, means that the effort in hygiene may be focused on visible cleanliness at the expense of practices that reduce microbial risk but are not visible to customers, such as handwashing between tasks, and correct raw food separation.

The irregular nature of health authority inspections that were reported across all three sites is consistent with the results by Botha et al. (13), who found the failure of routine monitoring to be one of the systemic weaknesses of the Ghanaian food safety system. The prevalence of blood testing and health card renewal as the predominant type of regulatory activity, instead of systematic evaluation of vending conditions, reflects what Monney et al. (12) characterized as implementation weakness at the community level despite a developed legal framework under the Public Health Act 2012 (Act 851). Adaku et al. (14), in a qualitative study in Greater Accra, for instance, found that the informal vendors were largely unaccountable to the formal food safety system, and the non-existence of trust between the vendors and the regulators was a central barrier to compliance. The concern that inspectors negotiated with vendors who were not in compliance, rather than enforcing rules directly, mirrors that finding. Where enforcement is negotiable, the deterrent value of inspection is lost, and vendors who do not comply have no meaningful consequence. The broad consensus among vendors that more frequent and stricter enforcement was needed implies that such a demand for consistent regulation exists among vendors themselves. The findings thus indicate that public health authorities in all three communities should be taken seriously.

The focused design of this study, which combines in-depth interviews and field observations, enabled a degree of contextual understanding of vendor behavior that structured surveys cannot provide. Triangulation of interview and observational data contributed to the credibility, especially in defining the discrepancy between the reported and the observed hand hygiene practice. However, there are several methodological limitations. The convenience sample of 20 vendors precludes generalisability to broader populations of vendors at the individual sites, and those vendors who declined or were not available at the time of approach may have different practices. Reliance on self-reported data on handwashing and regulatory compliance may be subject to social desirability bias, as vendors may have described ideal and not actual behavior. Field observation periods of 30 to 60 min only represent a segment of the working day and may not represent all conditions at a site. No microbiological testing of food or surfaces was undertaken, so the risk of contamination cannot be quantified. Despite these limitations, triangulation of data sources and preliminary theme checking with a sample of the participants provides confidence in the findings. While results are specific to Asamankese, Adabraka, and Kpando Gabi, the patterns obtained are similar to those found in similar informal food vending contexts in other parts of Ghana and sub-Saharan Africa, implying that the study is relevant beyond the three sites.

### Recommendations

Based on the findings of this study, some specific interventions are proposed for the Environmental Health and Sanitation Units of the concerned district assemblies, the Food and Drugs Authority and the local health directorates operating in Asamankese, Adabraka and Kpando Gabi. Health authorities should make regular, site-based inspection visits that evaluate actual vending conditions, such as hand washing facilities, food storage, waste management, and use of protective clothing, rather than restricting contact from regulators to blood testing and health card renewal. Food hygiene training programmes should be conducted at vending locations, in local languages and should include practical demonstrations tailored to the specific practices for which gaps were identified, including handwashing between tasks of different nature, proper separation of food and money, correct use and maintenance of protective clothing and prevention of cross-contamination. Local government assemblies should consider addressing the infrastructure gaps that limit the ability to comply with hygiene, such as providing cover for dustbins, access to clean water and improving drainage, especially in Kpando Gabi, where infrastructure gaps were most visible. Vendors should be provided with low-cost protective supplies like gloves, head covers, and food net covers, as the affordability of these materials was cited as a practical barrier. Enforcement needs to become consistent and consequential: inspectors need to impose sanctions in cases of non-compliance, and not to make deals with vendors who fail to meet food safety standards, because the practice of informal resolution currently being used undermines the deterrent effect of regulation. Finally, authorities should create a routine health surveillance system for the vendors that extends beyond annual blood testing to include periodic monitoring of vending site conditions as well as documentation of trends in hygiene practices across all three communities.

## Conclusion

This study has shown that food vendors in Asamankese, Adabraka, and Kpando Gabi had a working knowledge of food hygiene and were able to identify diseases linked to a lack of good practice, but there was a consistent and observable gap between what food vendors said and what was observed at their vending sites, especially in handwashing, protective clothing, and cross-contamination. Waste management, pest control, and food storage practices were as much dictated by available infrastructure and resources as they were by individual knowledge, with Kpando Gabi vendors being the most constrained. Health authority enforcement was sporadic in all three communities, and was mainly aimed at blood testing and health card issuance, but not comprehensive site assessment, and entry requirements for food vending were not always communicated. These findings highlight that an improvement in hygiene practice within this setting requires more than education alone, but the consistent enforcement of this education, provision of basic hygiene infrastructure, and locally appropriate education focusing on the practical conditions under which vendors work. Health authorities and local government assemblies in all three communities should ensure that vendors are given the means, and also the knowledge, to meet required standards, because the protection of the health of the consumers who depend on street-vended food is both a public health priority and a responsibility of the institutions mandated to regulate food safety at the community level.

## Figures and Tables

**FIGURE A 1:**
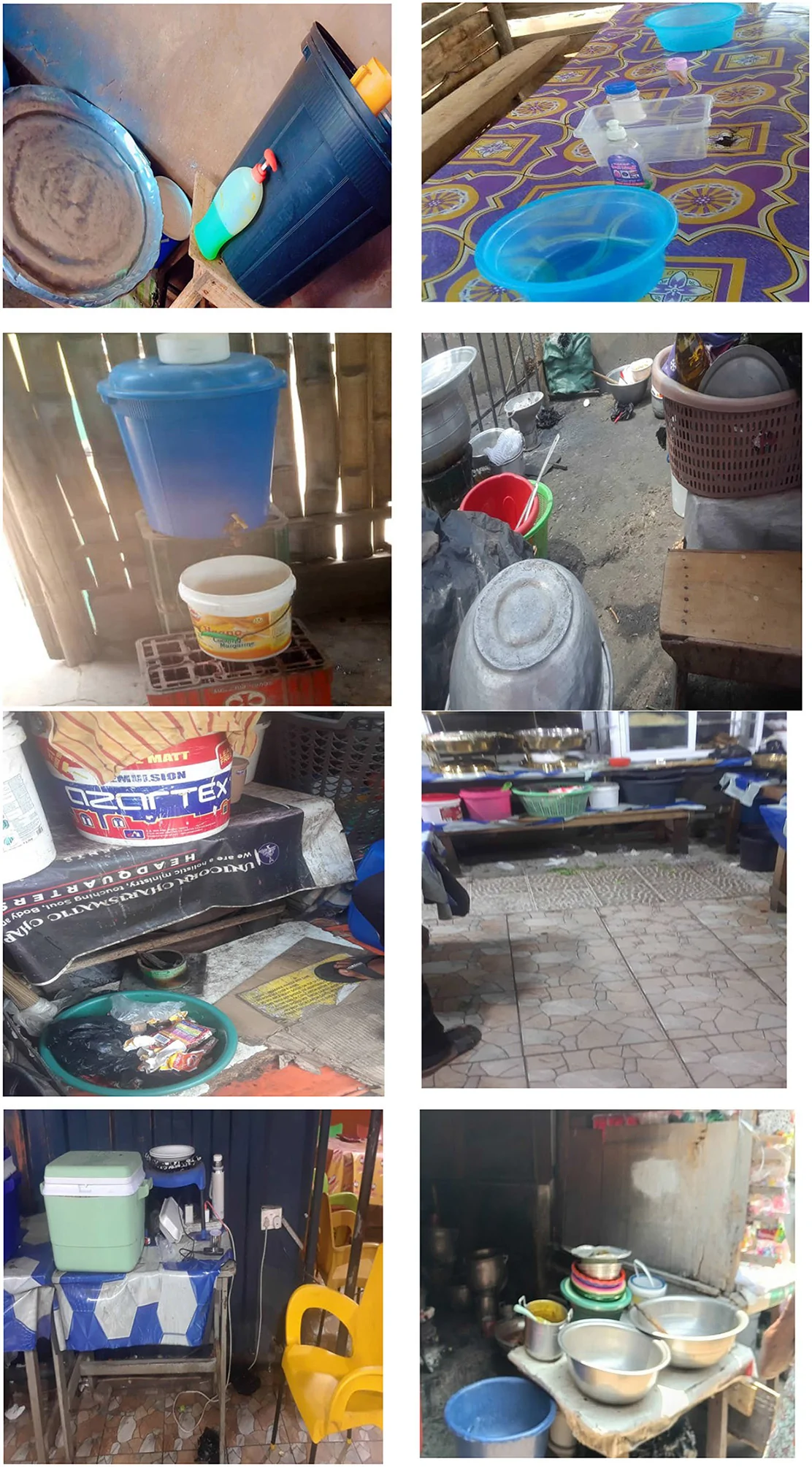
Pictures captured by the researchers in the food selling locations in Asamankese, Adabraka, and Kpando Gabi in the context of the structured field observations. Images depict water storage containers such as open drums, loosely covered basins; utensils stored in the open on wooden shelves and surfaces; a refuse bowl with food waste located near a food preparation area; a wet floor surface near food storage at one Adabraka site; and a vendor refrigeration setup at an Adabraka site.

## Data Availability

The raw data supporting the conclusions of this article will be made available by the authors, without undue reservation.

## Appendix

### A.1: Photo Gallery

The pictures below were captured by the research team during structured field observations in vending sites in Asamankese, Adabraka and Kpando Gabi. They record physical conditions at a number of sites, such as water storage techniques, utensil storage and handling, waste management, and the general state of the food preparation environment. These pictures offer pictorial support on the observational results mentioned in the Field Observation Findings section and complement the qualitative descriptions of the participants in the interview process after seeking consent from the participants.
